# Impacts of intellectual property provisions in trade treaties on access to medicine in low and middle income countries: a systematic review

**DOI:** 10.1186/s12992-019-0528-0

**Published:** 2019-12-30

**Authors:** Md. Deen Islam, Warren A. Kaplan, Danielle Trachtenberg, Rachel Thrasher, Kevin P. Gallagher, Veronika J. Wirtz

**Affiliations:** 1 Global Development Policy Center, Boston University, Boston, USA; 2 Boston University School of Public Health, Boston, USA; 3 Department of Global Health, 801 Massachusetts Avenue, 3rd floor, Boston, MA 02118 USA; 4 the Inter-American Development Bank, Boston, USA

**Keywords:** Access to medicines, Trade treaties, Low and middle income countries, Intellectual property, Systematic review

## Abstract

**Background:**

We present a systematic review describing ex-ante and ex-post evaluations of the impacts of intellectual property provisions in trade treaties on access to medicine in low and middle income countries. These evaluations focused on multilateral and bilateral trade agreements. We ascertained which IP provisions impacting access to medicines were the focus of these evaluations. We provide a further research agenda related to investigating the effect of trade agreement’s intellectual property provisions on access to medicines.

We followed systematic review guidelines with 7 different databases to identify post-2000 ex ante and ex post evaluations of trade treaties on access to medicines in low and middle-income countries. We included only quantitative ex-ante studies that used structural modeling and simulations to derive quantitative predictions and ex-post studies that utilized empirical data and econometric techniques to quantify the effects of intellectual property provisions in free trade agreements on host country’s pharmaceutical industry.

The search strategy identified 744 titles after removal of duplicates. We identified 14 studies that fulfilled all eligibility; 7 studies are ex-ante and 7 are ex-post. The studies looked at medicine price and cost, affordability, welfare effects and speed of medicine market launch. Changes in intellectual property policy due to the implementation of trade agreements affect price, medicines expenditure and sales, consumer welfare, and ultimately the affordability, of medicines. The direction and magnitude of the price effects differ between ex-ante and ex-post studies. Further, the reported impacts of policy changes due to trade agreements on medicine access seem clearly multifactorial.

**Conclusion:**

Both ex ante and ex post methods have advantages and limitations and, on balance, both types report, for the most part, an increase in price and a decrease in consumer welfare with imposition of intellectual property protection in trade agreements. The main differences between these studies are in the magnitude of the changes. There is a gap in our empirical understanding of the mechanisms through which such changes affect access to medicines and which outcomes relevant to access are most affected by which type of changes in intellectual property policy and law.

## Background

Intellectual Property (IP) provisions in free trade agreements (FTAs) ensure protection for the creation or invention of artistic works and goods, the creation or invention of which sometimes requires, as in the case of medicines, high sunk cost in the form of investment in research and development (R&D). Developing a new medicine requires large investment with high uncertainty. These R&D costs occur after a product patent is granted, which is typically very early in clinical development. IP provisions restrict the use and marketing of such goods and provide exclusive rights to the investors/creators to offset their sunk cost during clinical development [1]. This is to encourage more research and development (R&D) investment by the private sector to develop and invent new products [2]. Consequently, new or improved medicines are protected by patent and other IP provisions.

However, this protection creates a monopoly market for these medicines. Since the demand for medicines is generally price and income inelastic, this allows the owner of the patented medicine to charge a very high price [3]. As a result, there is growing concern among health care and development practitioners that IP provisions in trade agreements may have serious consequences on at least the affordability and/or availability of medicines in low and middle-income countries [4–7]. Affordability and availability of medicines are key dimensions of “access”.

The Agreement on Trade-Related Aspects of Intellectual Property Rights (or the TRIPS Agreement) set the standards for intellectual property protection in the world. It came into force on 1 January 1995 and is binding on all members of the World Trade Organization (WTO) [8]. The TRIPS Agreement sets minimum standards in the international rules governing patents, including patents on medicines [8]. Countries that are members of the WTO agree to these minimum standards in the way they enact and implement their patent laws. In recent years, many countries have been coming under pressure to enact or implement additional conditions in their patent laws than may negatively impact access to medicines – these are commonly known as ‘TRIPS-plus’ provisions [9].

There are TRIPS IP requirements and TRIPS-plus provisions that may negatively impact access to medicines, a list of which collectively might include: (1) relaxed standards of patentability, including patents on new uses, modifications of active pharmaceutical ingredients, new formulations/dosages [10] (2) patent term extensions to compensate for delays in patenting and registration decisions [11]; (3) limiting or eliminating patent oppositions [12]; (4) data/marketing exclusivity [12]; (5) patent/registration linkage [10]; (6) TRIPS-plus restrictions on compulsory and government use licenses [10]; (7) enhanced IP enforcement and remedies [10–12].

Providing a protected monopoly market to pharmaceutical products in countries could adversely affect access to originator medicines as well as to less expensive generic equivalents. Given this theoretical expectation of negative effects of stronger IP protection- e.g., TRIPS-plus, on access to medicines, a number of studies have been carried out to attempt to quantify the size of the effect. These studies are either *ex-ante* or *ex-post* in nature. *Ex-ante* studies use structural models and simulations to predict the likely impact of IP provisions on access to medicines, whereas *ex-post* studies utilize empirical data to measure the size of the effect. Some authors have suggested that the *ex-ante* studies invariably predict a robust negative effect of stronger IP regime on the affordability in the form higher prices or costs of medicines and availability in the form of lower consumption of medicines, whereas *ex-post* studies find mixed results from relatively mild negative to some positive effects [9].

Recently, Gleeson et al. [13] examined four trade and investment treaties to identify a channel of potential impacts of the specific treaty language on access to medicines and discussed studies that support their proposed analytical framework of pathways. They discussed the impacts on access to medicine mostly with respect to high-income countries and included mostly qualitative studies [13]. Our review is a complement to Gleeson et al. [13] as we focus more on quantitative empirical studies, and especially critically appraising the methodologies of these studies. Hence, the objectives of our study are to systematically review the literature for quantitative evidence that explore how the IP provisions in bilateral or multilateral FTAs affect the access to medicines in low and middle-income countries. Here, we have conducted a systematic literature review to analyze differences in methodologies of the studies, to summarize the range of impacts of IP protection on access to medicines and to assess the limitations of the studies. To this end, this systematic review attempts to answer the following questions:

1. What are the quantitative effects of different IP provisions in multilateral and bilateral trade agreements on access to pharmaceutical products in low and middle-income countries?
2. Which IP provisions are the main drivers of the effects on the different outcome variables measuring various aspects of access to medicines? Is there a cross-country variation in the effects of IP provisions?
3. What is a further research agenda related to investigating the effect of trade agreement IP provisions on access to medicines?

## Methods

We followed the Preferred Reporting Items for Systematic Review and Meta-Analysis (PRISMA) guidelines [14].

### Eligibility criteria

- Criteria 1 - Study design: We included only quantitative *ex-ante* studies that used structural modeling and simulations to derive quantitative predictions and *ex-post* studies that utilized empirical data and econometric techniques to quantify the effects of IP provisions in FTA on the importing country’s access to pharmaceuticals.
- Criteria 2 - Countries: We included studies that estimated the effects for low and middle-income countries. We used the World Bank classification to identify the low and middle-income countries [15].
- Criteria 3 - Time: We only considered post 2000 studies for inclusion. We note that the dateline to implement IP provisions under WTO’s TRIPS agreement is no later than 2000 for all countries except certain low and middle income countries. Most of the TRIPS-plus provisions in different bilateral FTAs are also a post-2000 phenomenon, for example the US-Jordan FTA (2000) and the US-Chile FTA (2004).

### Information sources

Between February and March 2019, we developed literature search strategies using key words related to IP provisions, access to medicines, and targeted countries. Initially, we used the title “Impacts of IP provisions in trade treaties on the access of medicine in low and middle income countries” in databases: AB/I, PubMed, Web of Science, Hein Online, JSTOR, Google® scholar and Econlit.

We developed a primary list of key words and PubMed MeSH terms, which we used in our comprehensive search for relevant studies. Search terms used in combination with the above-identified databases are shown below in Table 1, organized as Population, Intervention, Comparison and Outcome (PICO) components. All titles were reviewed, those outside the topic area of interest were deleted.

**Table 1:** Search terms organized as Population, Intervention, Comparison and Outcome items

Category (AND)	MeSH terms/Key words (OR)
Population	Developing Countries (MeSH), Low income countries, Least Developed Country (LDC), Middle income country
Intervention	Free Trade Agreement, Trade treaty, TRIPS/TRIPS-plus IP/Intellectual Property Right (IPR) (MeSH), Patent Data exclusivity/protection
Outcome	Medicine/Medicine Costs, Health Services Accessibility, Essential/supply & distribution, Access to medicines, Average/market price, Pharmaceutical Preparations/supply & distribution.

### Search results and selection process

A review team initially screened the titles and abstract from the first round of identification of relevant studies. At this stage, duplicate studies and studies that did not meet any of our pre-specified eligibility criteria were removed. Additional file 1 is a Table listing the combinations of search strings and the intial number of “hits”.

### Data items

We extracted information from the selected studies using topic domains and the framework for extraction is shown in Additional file 2. The main data item extracted from the studies is the outcome variables, which measures the various aspect of access to medicines. In most of the studies, the outcome variables are prices or costs and quantity or sales volume of medicines.

In addition to these outcome variables, some studies used time lags in new medicine launch or lags in different welfare measures as outcome variables. The key control variable in most of the studies is the time needed to capture the effect of moving from a weaker to a stronger IP regime. Thus, the comparison groups are observed or estimated effects of outcome variables before and after stronger IP implementation.

Other data items extracted from the studies are objectives of the studies, different information on country and medicine, types of IP provisions analyzed in the studies, key findings/results, recommendations. Detailed information on various studies are shown in Table 2 (*ex ante*) and Table 3 (*ex post*).

**Table 2:** Summary table of *ex-ante* studies

Studies (1)	Objective (2)	Methodology (3)	Country and medicine(s) studied (4)	Sample size (5)
Chaudhuri, Goldberg, & Gia [16]	To investigate the impacts of pharmaceutical patents for quinolones on prices and welfare in India	Two stage budgeting framework. Outcomes: medicine prices, consumer and social welfare. Comparison groups: sub-segments of systemic anti-bacterials medicine prices before and after implementing stronger patent laws.	Country: India, Medicines: quinolone sub-segment of anti-infectives.	Sample: 300 largest firms, representing roughly 90% of domestic retail sales. Range: January 1999 to December 2000.
Dutta [2]	To simulate the changes in consumer surplus, profits, market prices, and market quantities that would result from patent enforcement.	Structural model of demand, supply and entry. Outcomes: medicine prices, consumer and social welfare. Comparison groups: all pharmaceutical product prices before and after implementing stronger patent laws.	Country: India, Medicines: All pharmaceutical products sold in India.	Sample: The sample covers approximately 90% of all pharmaceutical sales in India; Range: 2001 to 2003.
Akaleephan et al [17].	To quantify the impact of TRIPs-plus provisions, especially the extension of market exclusivity of innovative medicines, in the proposed Thailand-USA FTA on medicine expense and medicine accessibility.	Simulation framework. Outcome variable: cost savings Comparison groups: generic medicines and innovative medicines under 10 years data exclusivity.	Country: Thailand; Medicines: 1136 International Non-proprietary Name (INN) of imported medicines.	Sample: 74 items out of 1136 INN; Range: 2000–2003
Azam [18]	To analyze the effects of TRIPS compliance on the prices, affordability and accessibility of pharmaceuticals in Bangladesh.	Use of different methods to analyze the different research questions posed in the paper: (a) doctrinal research for regulatory effects of TRIPS on pharmaceutical industry, (b) surveys, (c) case studies and interviews to analyze the expectation and perception regarding price, availability, affordability, etc., by the different stakeholders.	Country: Bangladesh; Medicines: All pharmaceutical products in Bangladesh.	Sample: 22 CEO interviews, top 20 medicines sales and top 10 medicines prices for time trend analysis. Range: Interview is from 2008, sales from 2008 to 09, price data for 1981 and 1991–92, and retail price survey for 2008–09.
Chaves et al. [19]	To assess the impact of TRIPS-plus measures as outlined in Mercosur-EU FTA on the public health in Brazil, especially on the public procurement of medicines.	Intellectual Property Rights Impact Aggregate (IPRIA) Model Outcome variables: public expenditures, domestic sales of medicines in Brazil, The current Brazilian market is used as a base for the calculations.	Country: Brazil. Medicines: HIV/AIDS and Hepatitis C.	Range: ARV medicines if from 2008 to 2015, Hepatitis C medicines is from 2006 to 2016.
Kessomboon et al. [20]	To measure the effects of US-Thai FTA on the access to medicines.	Model of Impact of Changes in Intellectual Property Rights (MICIPR) developed by Joan Rovira and jointly produced by the World Health Organization and the Pan- American Health Organization (WHO/ PAHO Region) Outcome variables: Different scenarios of patent extension and data exclusivity periods under the TRIPS-plus agreement.	Country: Thailand. Medicines: all active ingredients.	Range of projection is from 1992 to 2042,

**Table 3:** Summary table of *ex-post* studies

Studies	Objective	Methodology	Population	Sample data
Abbott et al. [21]	To assess the impact of stronger intellectual property protection in Jordan on the access to medicines	Mean and frequency comparison. Outcome: lag years in launching new medicines. Comparison groups: difference in years of lag in launching new innovative medicines in Jordan before and after the US-Jordan FTA.	Country: Jordan; Medicines: 46 essential medicines.	Sample: a sample of 29 of 46 essential medicines; Range: 1999 and 2004, pooled cross-section
Alawi & Alabbadi [22]	To analyze the effect of data exclusivity on the pharmaceutical sector in Jordan before and after the implementation of data exclusivity.	Trend analysis Outcome variables: prices, sale values, sale volume and sales Comparison groups: generic medicines, only originator medicines, originator to generic medicines, and generic to originator.	Country: Jordan; Medicines: all pharmaceutical products in Jordan.	Sample: a sample of 140 products representing 36.8% of total sales value in 2010. Range: 2004–2010.
Borrell [23]	To estimate the impact of patents on pricing of HIV/AIDS medicines in low and middle income countries in the late 1990’s.	Quasi-experimental study is used to study how the outcome variable differs for treatment groups and comparison groups that are not randomly assigned. Treatment group: all the country medicine pairs for which any ARV medicine is under a patent regime Comparison group: all the country-medicine pairs for which the medicine is not under a patent regime. Outcome variable: price	Country: Developing and least developed countries. Medicines: HIV/AIDS’ ARV medicines.	Sample: 21 developing and least developed countries with two groups of developing and low income countries, and 15 ARVs. Range: January 1995 to June 2000.
Duggan, Garthwaite & Goyal [24]	To estimate the effects of the 2005 implementation of a product patent system in India on pharmaceutical prices, quantities sold, and market structure.	OLS regressions Outcome variables: prices, sales volume difference specification and event study framework, where OLS regressions with patent dummy that takes value 1 in post patent regime and 0 in pre-patent regime are estimated, to investigate whether there is any statistically difference in log prices	Country: India. Medicines: All single molecule medicines	Sample: approximately 5100 Indian stockists. Range: 2003q1 to 2012q2.
Jung & Kwon [25]	To estimate the effect of stronger IPR on medicine access in low and middle income countries	Pooled cross-country multilevel techniques with subgroup analyses to identify factors both at country level and individual level that affect access to medicine and financial burden of purchasing medicines.	Country: all developing and least developed countries. Medicines: all medicines.	Sample: 35 countries, 660 to 38424households and 585 to 38,618 individuals. Range: 2002–2003.
Kyle & Qian [26]	To examines how TRIPS affects new medicine launches, prices and sales using data from 59 countries of varying levels of development.	Difference-in-difference estimation framework Outcome variables: speed of launch or new medicines, price, sales volume	Country: 59 countries of varying degrees of development.	Sample: 716 medicine-country pairs linked with patents; Range: 2000–2013 for prices and units sold and 1990–2013 for launch of new medicines.
Berndt & Cockburn [27]	To study the trade-off between stronger patent laws and early access to new medicines.	Survival analysis Outcome variable: sales volume, lag time of new medicine launch in India as compared to Germany and the U.S. due to Indian patent policies.	Country: India, Germany and USA; Medicines: new innovative medicines.	Sample: 184 new molecular entities approved by the US FDA. Range: 2000 to 2009.
Shaffer & Brenner [28]	To estimate the effect of IPR provisions in the Central American Free Trade Agreement on access to low price generic medicines in Guatemala.	Price comparison Outcome variables: Price Intervention group: Medicines purchased by both private and public sector in Guatemala of those that received data protection due to IPR provisions in the CAFTA Comparison group: Brand or generic equivalents that have no data protection.	Country: Guatemala. Medicines: all medicines available through various public-sector health programs.	Sample: 730 medicines on the Open Contract list. Range: 2005–2007.

## Results

**Fig. 1:**
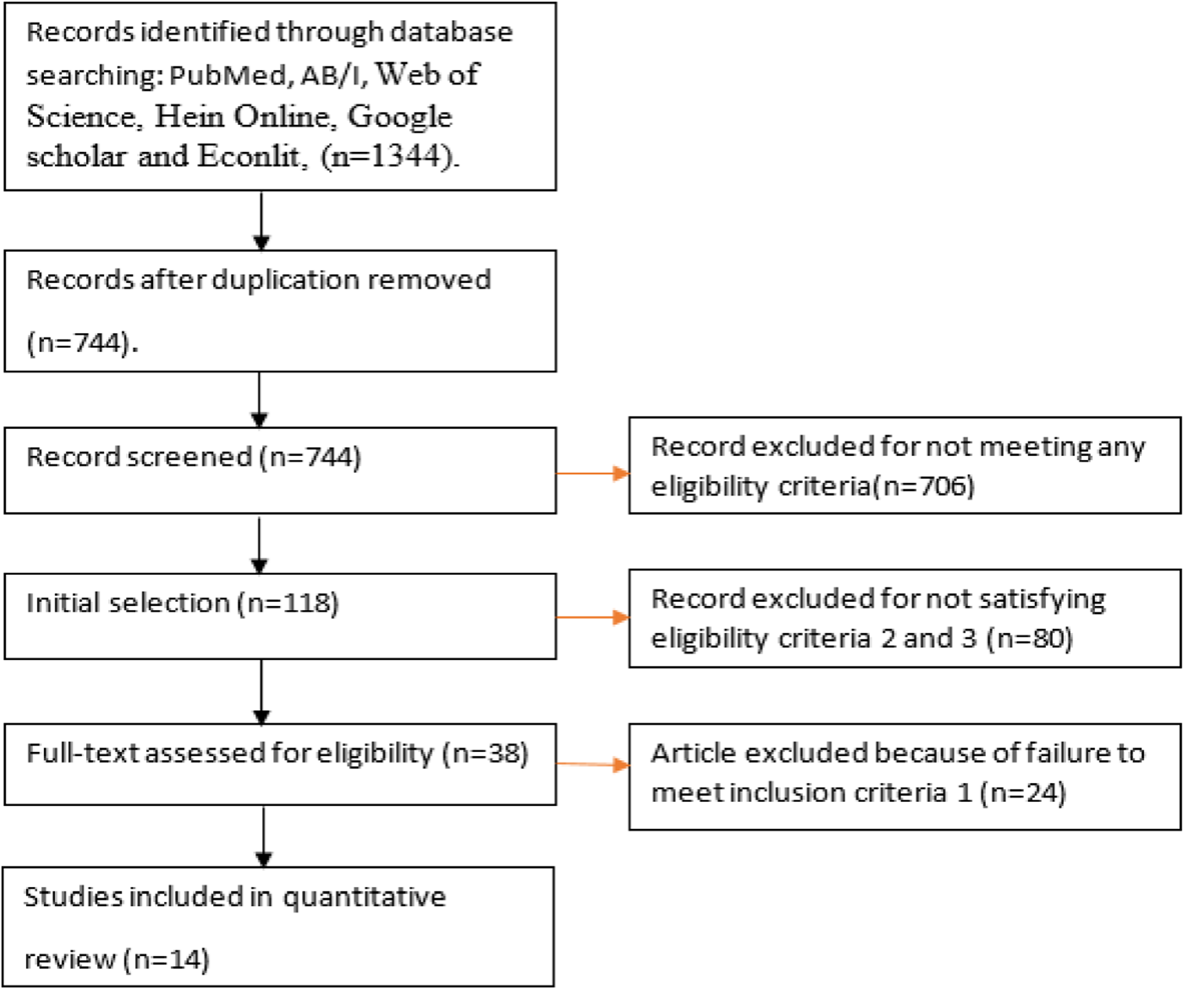
Selection process for the inclusion of studies

The search strategy identified 1344 unique abstracts to review (Fig. 1 and Additional file 1). After removal of duplicates, 744 titles remained. After the first stage review of the abstracts,118 studies were selected, the rest being excluded as not being relevant. At the second stage, we identified 38 studies (see References) that fulfilled eligibility criteria 2 and 3. Finally, three authors (DI, WAK, VW) independently reviewed all 38 studies selected from the second stage and out of the 38 studies, 14 studies are selected unanimously. Out of the 14 studies, 7 studies are *ex-ante* and 7 are *ex-post*.

The selection process of articles at different stages is shown below in Fig. 1.

Additional file 3 summarizes the rationale for exclusion of studies. Additional file 4 provides the authors’ checklist for determining study limitations.

Studies selected in our systematic review used a variety of methods to disentangle the effects of IP provisions on access to medicines.

Our overall results show that there are effectively only two broad IP categories for which different quantitative studies have attempted to estimate their impact on access to medicines. These are: a) the TRIPS Agreement, with implementation into national IP laws [2, 16, 18, 23–27], and b) TRIPS-plus provisions which include patent term extensions [19, 20] and data exclusivity or other commercial exclusivity provisions [17, 19, 21, 22, 26, 28]. Results of these studies shows that extending the patent term or ensuring data exclusivity has a larger negative effect on access to medicines compared with the IP benchmark set by the TRIPS Agreement [19, 20]. On the other hand, in comparing data exclusivity to patent term extension in Brazil, Chaves et al. [19] estimated larger expenditure on HIV and Hepatitis C medicine under data exclusivity than under patent term extension.

### Ex-ante studies

Chaudhuri et al. [16] used a two-stage budgeting framework (using data from 1999 to 2000) to investigate the effects on prices and welfare when one or more domestic generics are withdrawn from the quinolone market in India. Quinolones are a sub-segment of systematic antibacterials. Dutta [2] asked the same research question as Chaudhuri et al. [16] but for all pharmaceutical products and a more extended and updated data set with more control variables. Akaleephan et al. [17] simulated market share and prices of 74 International Non-proprietary Name (INN) imported medicines to estimate the potential cost savings in Thailand resulting from the absence of TRIPS-Plus provisions, particularly market exclusivity extensions, the lack of which would allow for increased price competition between innovative and generic producers.

Two papers used models of IP impact. Chaves et al. [19] used the Intellectual Property Rights Impact Aggregate (IPRIA) Model to project the impact of TRIPS-plus provisions of the Latin American Southern Common Market (Mercosur) -European Union (EU) FTA on the public expenditures and domestic sales of antiretroviral medicines (ARVs) and Hepatitis C medicines in Brazil. Kessomboon et al. [20] measured the effects of US-Thai FTA on access to medicines by using the Model of Impact of Changes in Intellectual Property Rights (MICIPR) to model the different scenarios of the patent extension and data exclusivity periods.

Two papers used trend analyses of medicines prices to predict the possible impact of IP provisions on access to medicines [18, 22].

### Ex-post studies

While all *ex-ante* studies included in our review are single country analyses, three of the seven *ex-post* studies are single country and four are cross-country analyses. Two of the three single country *ex-post* studies analyzed the impact of TRIPS-plus provisions in the US-Jordan FTA. Shaffer and Brenner [28] compared prices of medicines purchased by the public sector between 2005 and 2007 that received Central American Free Trade Agreement (CAFTA)-based IP data protection with their corresponding brand or generic equivalents that have no data protection to predict the effects of IP provisions on access to generic medicines in Guatemala.

Abbott et al. [21] compared the mean price and volume of 46 medicines before and after the US-Jordan FTA. Alawi and Alabbadi [22] used a time trend analysis to estimate the impact of specific TRIPS-plus clinical trial data restrictions in the US-Jordan FTA on prices, expenditure and volumes of different groups of medicines. Duggan et al. [24] used difference-in-difference analyses and a pre-post event study framework that takes into account other confounding factors to estimate the effects of the 2005 implementation of the TRIPS Agreement on the product patent system in India, specifically its impact on pharmaceutical prices, quantities sold, and market structure.

Kyle and Qian [26] used a cross-country difference-in-difference estimation framework to examine how imposition of IPs in the TRIPS Agreement are associated with new medicine launches, prices and sales using data from 59 countries of varying levels of development. Borrell [23] also used a difference-in-difference approach in a quasi-experiment framework to study the impact of IP provisions in various bilateral and multilateral trade agreements on pricing of certain ARVs in low and middle income countries and investigate how the pricing dynamics differ across different patent regimes.

Jung and Kwon [25] used a measurement of patent rights to grade the IP protection level of various countries and used regression analysis to examine the impact of IP protection and other country and household-level factors on access to medicines and the financial burden of purchasing …” medicines in low and middle income countries. Berndt and Cockburn [27] used time series analyses and focused on the impact of Indian IP policies measuring the delay in launch of new innovative medicines in India compared to Germany and the US.

### Measuring “access to medicines”

*Ex-post* and *ex-ante* studies used different measures of access to medicines to investigate how IP provisions can affect the various aspects related to access to medicines (see Table 4 (*ex ante*) and Table 5 (*ex post*)).

**Table 4:** Outcome variables, results and limitations of *ex-ante* studies

Studies	Main outcomes /dependent variables	Results	Examples of Limitations	
			Data	Method
Chaudhuri, Goldberg, & Gia [16]	Impact of patent enforcement on consumer surplus, profits and social welfare in the quinolone product market in India.	The total annual welfare losses to the Indian economy due to stringent patent laws in the quinolone sub-segment would be on the order of Rs. 20.16 billion or US$450 million.	Data range used to estimate the demand and supply parameters are from 1999 and 2000 and needs to be updated to obtain more accurate measures. The estimated demand elasticities are some proxies of real demand elasticities.	Elasticities are estimated using a reduced form demand system rather than estimating structurally. Further, assumes households are homogeneous in terms of income. Certainly, this assumption is not realistic
Dutta [2]	Impact of patent enforcement on prices, consumer surplus, profits and social welfare in 43 medicines market in India.	Profits for the patent holding firms increases from 0 to 3412% due to patent enforcement.	Data range used to estimate the demand and supply parameters are from 2001 and 2003 so data needs to be closer to 2005 to have better measure of elasticities and welfare.	Demand estimation does not include some important variable such as income of consumers. This implies that the elasticities are the same across different income levels, which is not a realistic assumption.
Akaleephan et al. [17]	Impact of data exclusivity on the accessibility, prices and total cost savings in 74 out of 1136 International Non-proprietary Name (INN) imported medicines in Thailand.	Consumption volume would be lower by 34.9% without generics	This paper uses only public sector data and since public sector might have higher bargaining power and costs of medicine for public sector are expected to very different from costs of medicine in private sector,	A simple linear regression is used to estimate market share following the generic entry. This simple linear regression would be biased due to omitting many important factors, which will also give biased estimates of cost savings.
Azam [18]	Impact of patent enforcement on sales, demand and prices in pharmaceutical sector in Bangladesh.	77% of surveyed executives of pharmaceutical firms either agreed (54%) or strongly agreed (23%) that the prices of medicines have increased and will go up further as a result of TRIPS compliance.	Data points used in trend analysis have at least 10 years’ interval, so by comparing price changes will not lend any sensible conclusion.	Trend analysis with large interval is not a rigorous way to find the effect of TRIPS provisions on medicine prices, many confounding factors can influence medicine prices.
Chaves et al. [19]	Impact of patent extension and data exclusivity on expenditure of HIV/AIDS and Hepatitis C medicines in Brazil.	Under the scenario that patent extension and 8 years’ data exclusivity are both adopted as proposed by EU, the ARV expenditure will increase by 69% and the expenditure on Hepatitis C will increase by more than 3000% in 35 years.		Simulations of alternative scenarios only considers the present average growth rate of expenditure on these two types of medicines, however, other factors such as change in demographics and disease prevalence rate would significantly affect the expenditure on these medicines.
Kessomboon et al. [20]	Impact of patent and data exclusivity extension on quantity and expenditure of all active ingredients in Thailand	Combining 10 years of patent extension with 5 years of delayed generic entry due to data linkage and 10 years of delayed generic entry due to data exclusivity will increase price index by 67%, expenditure to 23.6 billion USD over a 20 year period.	Outcome variable ‘expenditure’ is calculated by assuming a constant annual consumption growth of 12%, using actual expenditure for all the available years to calculate consumption growth would increase the precision of expenditure projection.	Projection does not consider other factors such income or population growth or change in demographics that might significantly change demand for medicines and hence the prices.

**Table 5:** Outcome variables, results and limitations of *ex-post* studies

Studies	Main outcomes /dependent variables	Results	Examples of Limitations	
			Data	Method
Abbott et al. [21]	Impact of market exclusivity and trade data protection on the number of generics, aggregate sales, and average price per daily dose for 29 essential medicines in Jordan.	Total cost of medicines increases from 81 million USD in 1999 to 125 million USD in 2004, a 53% increase in the total cost of medicines. Adjusting for increased sales volume and inflation, this represents an increase of 17% in the total cost of medicines.	Data used in this paper is for the year 1999 and 2004 and Jordan ratified new patent law in December 1999, registration of generic medicines may be artificially high in 1999 before the new patent law had become effective.	Comparing mean difference is a poor way to ascertain the rise in prices or costs to IPR provisions as equilibrium prices and quantity are influenced by many factors such as domestic and local economic factors, demographics, etc.
Alawi & Alabbadi [22]	Impact of data exclusivity on sales and cost saving in all pharmaceutical products in Jordan.	Following the expiration of data exclusivity, the medicine prices fall by about 56%.	Data is lacking both before and after the policy change. There is not even enough data for the trend analysis.	Trend analysis is not appropriate for causal analysis of data exclusivity. Outcome variables in this case are generally upward trending due to growth in population and disease prevalence and not due to policy change.
Borrell [23]	Impact of patent enforcement on pricing of HIV/AIDS’ ARV medicines in developing and least developed countries.	Medicine bundles containing at least one original medicine in a patent regime are on average priced 70% higher than medicine bundles containing only local copies marketed in no patent regimes.	Countries where there were patent laws in the pre 2000 era may not have same economic conditions and so treatment and control groups may also significantly differ in other dimensions in addition to patent policy.	Calculating price as sales divided by quantity is a very poor measure of actual prices or costs borne by the patients as HIV/AIDS medicines are publicly provided in developing countries.
Duggan, Garthwaite & Goyal [24]	Impact of patent laws on the average price, number of daily doses, and the number firms in India.	A small, negative, and statistically insignificant decrease (5.4%) in the quantity sold following a patent approval of a medicine		Regression analysis does not control for any economic or demographic variables that might significantly affect the outcome variables, prices, sales, quantity sold.
Jung & Kwon [25]	Impact of patent enforcement on the access to prescribed medicines and catastrophic medicine expenditure in developing countries.	A higher level of IPR protection is associated with higher probability that patients could not get access to their prescribed medicines.	Sample used in the analysis is from 2002 to 2003, when TRIPS implementation was not binding for the developing countries. Lack of access to medicine before 2005 cannot be attributed to IPR protection for most of the countries in the sample.	GP index is a poor measure of IPR protection as it does not consider the actual level of implementation of IPR laws.
Kyle & Qian [26]	Impact of patent and data exclusivity on launch speed, price level and quantity sold of medicines in 59 countries of varying degrees of development.	Products with expired patents sell in lower quantities and at lower prices than those that are on patent, but at higher prices and quantities relative to those that were never protected.		Difference-in-difference framework may not be a good framework to measure the effects of IPR on access to medicines as control and treatment groups of countries are very different.
Berndt & Cockburn [27]	Impact of patent laws on the difference in launch dates of new medicines in India, Germany and USA.	Almost one-quarter of the sample medicines were not available in India within the 10 years of their worldwide launch.	Launch date of medicines in a country is estimated by sales rather than using the official data of medicine approval.	No analysis is conducted to show that the difference in launch lag is due to IPR. The launch lags seem to be same before and after 2005 and so launch lag might be driven by other factors, which are not controlled in this study.
Shaffer & Brenner [28]	Impact of data protection on prices of all medicines provided through public sector health programs in Guatemala.	Medicine prices under data protection increase by 342 to 846% compared to equivalent generic medicines.	Only uses data of prices of public sector medicines, but prices could be higher or lower in private sector.	This paper uses trend analysis, but it does not test any structural break due to data protection and hence simply calculating changes in prices over time cannot be entirely attributed to the change in policy regime.

#### Price and cost

Most of the *ex-ante* studies found large negative effects of stronger IP provisions on prices and costs of medicines. Following the introduction of stronger IP laws, prices of medicines were predicted to go up by 50% to over 600% [2, 16, 18, 20, 28].

On the other hand, the majority of *ex-post* studies IP found price increases ranging from 3% to about 50% after the adoption of IP provisions found in the TRIPS Agreement itself and in TRIPS-plus FTAs [21–24], while some other found a small decrease in prices [25, 26].

Some *ex-ante* studies estimated changes in expenditures due to changes in IP regime. The *ex-ante* study of Akaleephan et al. [17] estimated the costs of data exclusivity to range between USD 0.1 to 1.1 million per item in the first year and USD 4.4 to 26.9 million per item in the tenth year in Thailand, while Chaves et al. [19] found that public expenditure on ARVs will increase by about 70% because of TRIPS-plus provisions as outlined in the Mercosur-EU FTA. Similarly, Kessemboon et al. [20] obtained the additional expenditure on medicines due to implementing US-Thai FTA provision, which ranged from over 11 billion USD to 23 billion USD for a 20-year period, under different combinations of patent term extensions and data exclusivity periods.

#### Availability of medicines

Few studies estimated the effects of changes in IP provisions on the availability and quantity of medicines consumed. Akaleephan et al. [17] predicted that the consumption volume would be about 35% lower without generics due to the data exclusivity provision in the proposed US-Thailand FTA. In contrast, the *ex-post* study of Kyle and Qian [26] found that products in TRIPS-compliant countries with expired patents, were sold in lower quantities than those products that are on patent, but at higher quantities relative to those that were never patent protected. Similarly, the *ex-post* study Duggan et al. [24] estimated a small, negative, and statistically insignificant decrease (5.4%) in the quantity of medicines sold following the imposition of a TRIPS-based product patent system in India.

#### Welfare effects

A few *ex-ante* studies estimated the welfare effects of a stronger IP regime. Chaudhuri et al. [16] estimated that the total annual welfare losses to the Indian economy from the withdrawal of generics in the market of quinolone sub-segment would be on the order of US$450 million. Similarly, Dutta [2] estimated the total loss to the consumer from patent enforcement and price deregulation in the market of 43 medicines in India to be $378.5 million and this decrease in consumer welfare would be significantly ameliorated in the presence of price regulation. This means that patent monopolies are less effective as price increasing agents if the government actually regulates prices.

#### Launch delay

Some *ex-post* studies estimated the delay in launching of new innovative medicines due to no or weak IP protection. Berndt & Cockburn [27] found that during 2000–2009, the estimated median launch lag was 4.5–5.0 years in India, compared to about a year in Germany and less than 2 months in the United States. They found that more than half of the medicines that became newly available in India during 2000–2009 were made and sold by multiple manufacturers within 1 year of their introduction and they suggested this was due to “weak patent protection”. Kyle and Qian [26] found that on-patent products were the most likely products to be launched and medicines that were never patented are unlikely to be launched at all, regardless of per capita income of countries.. They found countries with higher per capita income have more product launches of on-patent medicines compared to medicines with expired patents. Kyle and Qian [26] therefore asserted, in effect, that medicines are more likely to be marketed if they are protected by post-TRIPS patents.

## Limitations of the studies

We identified several limitations of both *ex-ante* and *ex-post* studies with respect to methodologies and data used in those papers. The main limitations of each paper regarding data and methodologies are shown in the Tables 4 and 5. Here we discuss limitations of studies in details.

### Unknown factors influencing outcome variables

Some studies [18, 21, 22, 27, 28] that used time trend or time difference of various measures of access to medicines cannot claim for certain that the changes in the outcome variables, such as prices, costs, availability of medicines, were due to changes in the IP regime. Many factors, such as changes in demographics, disease prevalence, and economic growth, may have affected those outcome variables. Studies that used two-stage budgeting [16] or structural estimation [2] controlled for many factors, but yet there are many individual or household characteristics such as age, gender, ethnicity, family size, income, residence, and the like are not accounted-for in these estimations. Studies that used market share data, e.g. [18, 21, 23, 27, 28], do provide information on those variables. However, demand for medicines is not the same as demand for other goods and those neglected micro level variables can be correlated over time and space, which may induce bias and inconsistency in the demand estimates. These studies also did not incorporate macro level variables such as changes in demographics, disease prevalence, economic growth, and the like which are correlated with the outcome variables and omitting these important factors will lead to incorrect welfare measure of the change in IPR regime.

Studies that used some specialized model, such as IPRIA [19], MICIPR [20], and that of Akalephaan et al. [17], also suffer from lack of controlling proper co-factors as these models are macroeconomic in nature. All these models used simulations based on common assumptions, i.e., constant growth rates of macroeconomic variables and generic and innovative medicines being perfect substitutes for each other.

In addition, all these studies ignore changes in public policies due to internal or civil society pressure in response to rise in cost of medicines. For example, pricing on some HIV medicines was significantly influenced by campaigns for discount pricing and voluntary licenses and by increases in donor funding for health, including medicines purchases [5]. Macroeconomic constraints on government budgets, and in particular health sector budgets, could have significantly impacted overall public spending on medicines; as well there could have been changes in government priorities for health spending [3]. Also, the nature of - and changes in- the health insurance sector could also affect purchasing/usage decisions [29]. There was as well a general failure to consider the timing effect of new IP regimes, as well as the level of IP protection that existed before the relevant study dates [30]. Analogously, medicine availability can be impacted by registration decisions, placement of medicines on an essential medicines list (with some such listing being delayed because of high prices and patent status), incorporation of medicines in relevant treatment guidelines, prescriber preferences, commercial marketing to prescribers, and a host of other factors [31].

Studies that used a difference-in-difference framework [23, 24, 26] did not provide discussion whether there were any concurrent trends between the outcomes of interest and similar trends in the control variables, and hence authors of those studies could not claim causality between the change in the policy regimes and change in outcome variables. In brief, the lack of control for many important confounders is a common limitation of all the included studies.

### Dataset limitations

Lack of an appropriate data set to test the proposed hypotheses is another important limitation of these studies. Chaudhuri et al. [16] used a data range only from 1999 to 2000 to estimate the demand and supply parameters with regard to quinolones. India strengthened her patent law in 2005, so these parameter estimates used by the authors may not be up to date and welfare estimations based on these estimates will likely not be accurate.

Akaleephan et al. [17] used only public sector data, but not private hospital or retail pharmacy or end user prices of medicines. In many settings the public sector has higher bargaining power and costs of medicines for the public sector are expected to very different from costs of medicine borne by the private sector. Berndt and Cockburn [27] used sales data to estimate the medicine sales and lags in product launch date in India, which may only reflect demand or supply side constraints rather than the policy constraint originating from patent policies. Indeed, although other concerns matter, companies product launch decisions largely based on regulatory and commercial prospects. For example, companies often delay launching in certain lower-price markets that are used for reference pricing by higher-price markets. In addition, barriers to market registration and timeliness of registration varies greatly between countries [32].

There are as well questions about whether companies have established marketing and distribution channels in particular countries [33].

Jung and Kwon [25] used a sample from 2002 to 2003 in their analysis, which is not a good sample as TRIPS implementation was not binding for the developing and least developed countries before 2005. So, lack of access to medicines before 2005 cannot be attributed to IP protection for most of the countries in the sample. They measured IP protection using the method of Ginarte and Park [34], which is a poor measure of IP protection as it does not consider the actual level of implementation of IP laws. We further note that the only *ex-post* studies in this review looking at the impact of TRIPS-plus provisions were done in Jordan [21, 22].

### Endogeneity: inappropriate or incorrect causation

Chaudhuri et al. [16] used the number of stock keeping units (SKU) as a proxy of prices for each product group and assumed that the number of SKUs in each product group are uncorrelated with the other factors influencing medicine demand. Clearly, this is questionable as entry or exit of different producers in the same product group depends on these very factors as actual prices are fixed in the market. This means that their key variable is still correlated with unobserved market properties. A similar problem occurs in Dutta [2] as the product level changes are likely correlated with unobserved/omitted variables, e.g., such changes are generally correlated with firms’ unobserved and/or omitted properties that are not able to be included in the analysis. For example, the market presence and molecule age of medicines made by competitor firms not only affect the market price of firms but also change the set of available options to buyers and hence affect the demand of medicine produced by the firm. Akaleephan et al. [17] used a simple linear regression to estimate market share following the generic entry. So, it is highly likely that the estimate of this simple linear regression would be biased due to omitting many important supply and demand side factors, which would lead to a biased estimate of costs savings.

### Unrealistic assumptions

*Ex-ante* studies in many cases used arguable assumptions in estimating or simulating the impact of IP provisions on access to medicines. Dutta [2] assumed that factors impacting consumer preference for a given medicine are independently and identically distributed, which allows one to derive the market share for each category of medicines. However, since the demand for medicines generally depends on the physician’s prescription, so preference for a particular medicine is unlikely to be independent across consumers. Thus, in the case of estimating the demand parameters of different medicines, the nested logit framework - which assumes that consumer preferences are independent across medicines - may not a suitable framework. Akaleephan [17] assumed that market for innovative and generics are perfectly competitive. However, the more appropriate market structure in this case would be oligopolistic as generics and innovative medicines are differentiated products. Kessomboon et al. [20] also used very stringent assumptions, such as constant price elasticities of demand and constant price differential of active ingredients under data exclusivity and price competition, and time-invariant market shares of the domestic and the innovative industry. These assumptions are not very realistic.

## Discussion

Our systematic literature review makes several contributions:

First, the studies we have reviewed show that changes in IP policy due to the implementation of trade agreements are associated with changes in price, medicines expenditure and sales, consumer welfare, and ultimately the affordability, of medicines. The direction and magnitude of the effects differ between *ex-ante* and *ex-post* studies. Regarding prices and costs of medicines, *ex-ante* studies predict that prices and costs (primarily public expenditure) of medicines could increase several hundred percent due to the impact of various IP provisions such as increased patent enforcement, TRIP-plus and other provisions in various multilateral and bilateral agreements. These *ex-ante* studies confirm what the theory would say [35] i.e., that stronger IP monopoly rights would tend to eliminate competition and thus incur societal costs which are higher prices for IP products.

On the other hand, empirical *ex-post* studies found at most a moderate increase in prices and costs of medicines due to the imposition of similarly heightened IP rules. There is, however, some consensus between *ex-ante* and *ex-post* studies that TRIPS-plus provisions relating to clinical data protection, rather than the imposition of more stringent patent rules, would cause a larger increase in prices and costs of medicines and lead to lower access to medicines. We note that extending the patent term may have an additionally important, but as-yet undifferentiated, impact since most data protection provisions are confined within the period of existing patent protection and are not additive to patent extensions. Second, the reported impacts of IP changes due to trade agreements on access to medicines seem clearly multifactorial. Duggan et al. [24] found an insignificant increase in medicine prices after patent law reform and argued that this might be because the existing generic producers are ‘grandfathered’ and continue to produce the generic medicines even after patent enforcement. This is because TRIPS does not require retroactive IP protection on pre-1994 medicines. Kyle and Qian [26] found that the existence of a patented molecule does not always block generic imitation, nor does the lack of patents always deter an originator from making a product available. They also pointed out that effects of IP may well be different depending on the size of the local generic sector, e.g., the impact in India with its large and robust generic medicine sector may be different as compared most other low and middle income countries. They asserted that the “… existence of IPs is neither necessary nor sufficient …” for the launch of pharmaceutical innovations at the country level. This suggests substantial heterogeneity in the effects of IPs, both across countries and across medicines.

Third, *ex-ante* studies that use structural models are often better able than *ex-post* studies to draw causal effects of IP policy changes on access to medicines. But *ex-ante* studies are based on stringent model assumptions and provide only counterfactual estimates. On the other hand, *ex-post* studies attempt to measure the actual effects of IP protection on access to medicines, but in most of the *ex-post* studies, the empirical models are not well identified and hence only a weak causal inference can be established. Shadlen et al. [30] emphasize the temporal impact of changes in IP provisions. The authors suggest that, depending on when countries first began allowing drugs to be patented, TRIPS-Plus provisions will have different effects.

To access the real effects of IP policy changes due to trade agreements on access to medicines, which approach, *ex-ante* or *ex-post*, would be more accurate? It is clear from our findings, that both methods have advantages and limitations and, on balance, what does seem clear is that both types of studies predict, for the most part, an increase in price and a decrease in consumer welfare with imposition of IP in trade agreements. The main differences between these studies are in the magnitude of the changes. The fact that such a difference in magnitude exists may well be due to the assumptions in *ex-ante* models and limitations of *ex-post* studies, but there are likely omitted and unmeasurable institutional variables in the health policy ecosystem that contribute as well.

Fourth, our literature review found that the impact of IP provisions in various trade agreements manifests itself through the healthcare/pharmaceutical ecosystem. Thus, this has implications for developing better empirical models to measure the effects on key outcome variables. For example, Jung and Kwon [25] assert that IP exerts an influence on medicine utilization only in countries above a certain income level. They did not observe any significant effect of IP on access to medicines in low-income countries where Gross Domestic Product (GDP) per capita is below 1000 US dollars. They also found that those who live in rural areas and have health insurance were more likely to report that they could not access their prescribed medicines compared to those who live in urban areas. Shaffer and Brenner [28] noted that CAFTA’s data exclusivity and patent rules were implemented in Guatemala through domestic law so one might ask whether differences in domestic implementation of IP provisions have an impact on the effect size on access to medicines. Kessomboon et al. [20] suggested that the strategies to address the negative consequences of an FTA that affect access to medicines would be based on several elements of the pharmaceutical system: medicine selection, procurement, distribution, and medicine use. Overall, we found a scarcity of studies analyzing the effects of changes in IP on different elements of the pharmaceutical system. Indeed, all of the *ex-post* studies on TRIP-plus provisions were done in Jordan.

Fifth, our literature review identified important research gaps that should be addressed: Is there a differential impact of IP provisions on different medicines for similar conditions, what is the impact of such provisions on essentially interchangeable medicines such as the insulins, what is the impact on local medicine production, on medicine quality, on affordability for various socio-economic groups, on medicine procurement, on medicine dispensing, on patient choice, and on prescriber choice in both public and private sectors? Indeed, our results suggest that we are unable, at this time, to unpack the main IP drivers that impact access to medicines. Further, the quantitative literature we have reviewed simply cannot say much about “cross country variation” in the effects of IP provisions on access to medicines. This is a clear research gap and should be subject to future research.

Clearly, trade treaties will manifest their impacts on a complex healthcare ecosystem. Sustainable Development Goal (SDG) 3.8 emphasizes the need for “access to safe, effective, quality and affordable medicines” [36]. Assessing the effect of IP provisions in trade treaties- by whatever methodology and setting aside methodological implications and limitations- should include a study of domestic implementation, access, availability and affordability; safety, efficacy and quality; rational use of medicines; procurement; and local production capacity.

Finally, several approaches and data sources would be relevant in this regard and should be driven by the research question, irrespective of methodology. The complex impact of IP and trade provisions on “access to safe, effective, quality and affordable medicines” calls forth relationships between medicines and health financing, human resources, health information and service delivery [31]. Studies looking at the impact of trade rules on populations’ access to medicines should no longer be addressed mainly through ‘siloed’ approaches that focus primarily on price. On one hand, mixed methods approaches can, in principle, offset the limitations of quantitative and qualitative studies by allowing for both exploration and analysis in the same study. Quantitative research is weak in understanding the context and qualitative research does not often lend itself to statistical analysis and generalization. For example, one possible next step in either *ex-ante* or *ex-post* studies is to use granular household level data, particularly in those many low and middle income countries where patients pay out-of-pocket for medicines.

For *ex-ante* studies, this will allow researchers to estimate the demand elasticities of medicines and hence, this will help predict the changes in prices and quantities, and effects on social welfare more accurately. For *ex-post* studies, granular medicine level data and rigorous empirical strategies could potentially isolate the causal effect of IP policy change on access to medicines directly at the patient level. This can be done in combination with qualitative research on perceptions of relevant stakeholders to changes in IP provisions and access to medicines. One disadvantage of the simplicity of *ex-ante* models is that it makes assumptions that fail to mirror the complexities of the relationships between variables in the real world, for example, the price differential of a product before and after going off patent, and the constant price elasticity of demand. It is usually difficult to estimate realistic values for key variables.

On the other hand, the challenges of dynamic complexity in the public health ecosystem may be effectively addressed with the modeling methodology of system dynamics. The methodology involves development of causal diagrams and policy-oriented computer simulation models that are unique to each problem setting [37]. The International System Dynamics Society was established in 1983, and within the society a special interest group on health issues was organized in 2003 [37]. System dynamics uses computerized models in which alternative policies and scenarios can be tested in a systematic way that answers both “what if” and “why.”

Our review has several limitations that should be taken into considerations. We limited our search to seven search engines, which may have resulted in missing relevant studies. We also did not perform a meta-analysis for data synthesis because of the variation in outcome variables chosen.

## Conclusion

Many people lack access to medicines, particularly in low and middle income countries, even without any IP protection laws. Imposing IP protection laws or strengthening these laws as a result of trade agreements may further reduce the access to medicines. The magnitude of the effect on different outcome variables such as price, medicines expenditure and consumer welfare differ depending on a host of factors, most importantly domestic policies in place to counteract the potential negative effects on access. More studies are necessary to fill the gap in understanding the mechanisms through which changes in IP affect medicines access and which outcomes relevant to access are most effected by which type of changes in the IP.

## Supplementary information

**Additional file 1.** Table of search terms and initial number of references found using these terms.

**Additional file 2.** Data extraction table.

**Additional file 3.** Reasons for not selecting a particular study.

**Additional file 4.** Checklist to identify study limitations.

## Data Availability

Data sharing is not applicable to this article as no datasets were generated or analysed during the current study.

## Abbreviations

ARVs: Antiretroviral medicines
CAFTA: Central American Free Trade Agreement
EU: European Union
FTA: Free Trade Agreements
INN: International Non-proprietary Name
IP: Intellectual Property
IPRIA: Intellectual Property Rights Impact Aggregate
IPR: Intellectual Property Rights
LDC: Least developed country
MeSH: Medical Subject Heading
MICIPR: Model of Impact of Changes in Intellectual Property Rights
PICO: Population, Intervention, Comparison and Outcome
PRISMA: Preferred Reporting Items for Systematic Reviews and Meta-Analyses
R&D: Research and Development
SDG: Sustainable Development Goal
SKU: Stock Keeping Unit
TRIPS: Trade-Related Aspects of Intellectual Property Rights
WTO: World Trade Organization
