# Supplementary material for: Impacts of intellectual property provisions in trade treaties on access to medicine in low and middle income countries: a systematic review
Source: Global Health. 2019 Dec 30;15:88. doi: 10.1186/s12992-019-0528-0 (PMC6937733; doi:10.1186/s12992-019-0528-0)
Supplement: Supplementary file 1 — Additional file 1. Table of search terms and initial number of references found using these terms. [file 12992_2019_528_MOESM1_ESM.docx]

**Additional Files**

**File name (Additional file 1.doc)**

**Table of search terms and initial number of references found using these terms**

| Combinations of search strings and search results | | | |  |  |
| --- | --- | --- | --- | --- | --- |
| Key words search | | Initial Search results | | |  |
| trade treaty AND developing countries OR low income countries AND drug cost OR access to medicine | 346 | |  |  |  |
| TRIPS/TRIPS plus AND Developing countries OR low income countries AND Drug cost OR access to medicine | 71 | |  |  |  |
| Intellectual Property AND Developing countries OR low income countries AND Drug cost OR access to medicine | 202 | |  |  |  |
| Patent AND Developing countries OR low income countries AND Drug cost OR access to medicine | 124 | |  |  |  |
| Data exclusivity AND Developing countries OR low income countries AND Drug cost OR access to medicine | 315 | |  |  |  |
| Compulsory licensing AND Developing countries OR low income countries AND Drug cost OR access to medicine | 286 | |  |  |  |
| Total | 1344 | |  |  |  |
