# Supplementary material for: Impacts of intellectual property provisions in trade treaties on access to medicine in low and middle income countries: a systematic review
Source: Global Health. 2019 Dec 30;15:88. doi: 10.1186/s12992-019-0528-0 (PMC6937733; doi:10.1186/s12992-019-0528-0)
Supplement: Supplementary file 2 — Additional file 2. Data extraction table. [file 12992_2019_528_MOESM2_ESM.docx]

**Additional Files**

**File name (Additional file 2.doc)**

**Data extraction table**

**Framework table for identifying and extracting information about each study**

| General Information | | | | | |
| --- | --- | --- | --- | --- | --- |
| First author: | Year of study: | | | Title: | |
| Objective: | | | | | |
| Publication type Journal Article ⬜ Report ⬜ Other (specify e.g. book chapter)___________________ | | | | | |
| Country/region of study: | | | | | |
| Funding source of study: | | | Potential conflict of interest from funding? Y / N / unclear | | |
| Type of FTA (bilateral/multilateral) | | | IP provisions: | | |
| Data source: | | | Data range: | | |
| Method/model to determine outcome: | | | | | |
| **Types of outcome measures** | | List of outcome variables: | | |  |
|  |  | Outcome measured at a country level or industry level? | | | Details: |
| Main findings/results: | | | | | |
| Limitations (including GRADE criteria): | | | | | |
| Conclusion: | | | | | |
