# Supplementary material for: Impacts of intellectual property provisions in trade treaties on access to medicine in low and middle income countries: a systematic review
Source: Global Health. 2019 Dec 30;15:88. doi: 10.1186/s12992-019-0528-0 (PMC6937733; doi:10.1186/s12992-019-0528-0)
Supplement: Supplementary file 3 — Additional file 3. Reasons for not selecting a particular study. [file 12992_2019_528_MOESM3_ESM.docx]

**File name (Additional file 3.doc)**

**Reasons for not selecting a particular study**

**A table showing the exclusion criteria and reasons why a particular article was not selected in the final review.**

| Studies | Reason for not including in the review |
| --- | --- |
| Ala (2013) [1] | Does not measure the effect of IP provision on access to medicines |
| Aksan (2013) [2] | Theoretical paper with no empirical analysis. |
| Attaran (2004) [3] | No empirical analysis on prices or quantity or any other measure of access to medicines |
| Attaran and Gillespie-white (2001) [4] | No empirical analysis. |
| Basant (2007) [5] | No empirical analysis. |
| Beall et al (2015) [6] | Comparison of two TRIPS-plus provision: Compulsory license and parallel import and no analysis of impact of TRIPS/TRIPS-plus provisions on access to medicines. |
| Boring (2010) [7] | Empirical analysis of pharmaceutical trade and patent protection and no analysis of patent or any other TRIPS provision on the access to medicines. |
| Correa (2006) [8] | No empirical analysis. |
| El-Said and El-Said (2007) [9] | No empirical analysis. |
| Bognar et al. [10] | No empirical analysis |
| Goldberg (2010) [11] | Elaboration of analysis of Chaudhuri |
| Hellerstein (2012) [12] | Not directly measures the impact of TRIPS/TRIPS-plus provisions on access to medicines. |
| Jani and Dholakia (2015) [13] | Not about IP and access to medicines. |
| Kumar (2003) [14] | Not about the impact of IP on access to medicine, rather discuss relationship between IP technology transfer and trade in pharmaceuticals. |
| Kyle and McGahan (2012) [15] | Not about IP and access to medicines but about IP and investment. |
| Malpani (2009) [16] | No empirical analysis. |
| Mazuka (2009) [17] | No empirical analysis. |
| Moir et al. (2014) [18] | Estimation based on other papers |
| Pascual (2014) [19] | No empirical analysis |
| Sakthivel (2005) [20] | This is a book chapter and it does not estimate the effect of IP on access to medicines and discuss overall access to medicines in India. |
| Smith, Correa and Oh (2009) [21] | No empirical analysis. |
| Stavropoulou and Valletti (2015) [22] | Theoretical paper and no empirical analysis. |
| Supperamaniam et al (2009) [23] | No empirical analysis and just compares prices of medicines in UK and Malaysia. |
| Townsend, Gleeson and Lopert (2016) [24] | No longer applicable as the USA has pulled out of TPP. |

References:

1. Ala MU. A firm-level analysis of the vulnerability of the Bangladeshi pharmaceutical industry to the TRIPS agreement: Implications for R&D capability and technology transfer. Procedia Economics and Finance. 2013; 5:30-9.
2. Aksan AM. Appropriate health R&D and intellectual property rights reform in developing countries. Economica. 2013;80:475-95.
3. Attaran A. How do patents and economic policies affect access to essential medicines in developing countries?. Health Affairs. 2004; 23:155-66.
4. Attaran A, Gillespie-White L. Do patents for antiretroviral drugs constrain access to AIDS treatment in Africa?. Jama. 2001; 286:1886-92.
5. Basant R. Intellectual property rights regimes: Comparison of pharma prices in India and Pakistan. Economic and Political Weekly. 2007; 29:3969-77.
6. Beall RF, Kuhn R, Attaran A. Compulsory licensing often did not produce lower prices for antiretrovirals compared to international procurement. Health Affairs. 2015;34: 493-501.
7. Boring A. Does Foreign Patent Protection Increase the United States' Trade of Pharmaceuticals with Developing Countries. Université Paris Dauphine (LEDa-DIAL) Ph D. thesis. 2010. Available at https://pdfs.semanticscholar.org/60cf/e01ced295a661c09451da6624f26a3d9f7d5.pdf or [https://www.etsg.org/ETSG2010/papers/Boring.pdf. Accessed 7 August 2019](https://www.etsg.org/ETSG2010/papers/Boring.pdf.%20Accessed%207%20August%202019).
8. Correa CM. Implications of bilateral free trade agreements on access to medicines. Bulletin of the World Health Organization. 2006;84:399-404.
9. El‐Said H, El‐Said M. TRIPS‐Plus implications for access to medicines in developing countries: lessons from Jordan–United States Free Trade Agreement. The Journal of world intellectual property. 2007; 10:438-75.
10. Bognar CL, Bychkovsky BL, Lopes Jr GD. Compulsory licenses for cancer drugs: does circumventing patent rights improve access to oncology medications? Journal of global oncology. 2016; 29:292-301.
11. Goldberg PK. Intellectual property rights protection in developing countries: the case of pharmaceuticals. Journal of the European Economic Association. 2010; 8:326-53.
12. Hellerstein R. What do drug monopolies cost consumers in developing countries?. Economics Letters. 2012; 116:108-11.
13. Jani V, Dholakia RH. Does Trade Make Asian Children Healthier? Indian Institute of Management No. 2015-10-08. 2015; 1-21. Available at https://www.semanticscholar.org/paper/Does-Trade-Make-Asian-Children-Healthier-Jani-Dholakia/23eada89d8b9646ffa8b2a4bce2b0bcf5a90920a. Accessed 7 August 2019.
14. Kumar N. Intellectual property rights, technology and economic development. Economic and Political Weekly. 2003; 38:209-25.
15. Kyle MK, McGahan AM. Investments in pharmaceuticals before and after TRIPS. Review of Economics and Statistics. 2012; 94:1157-72.
16. Malpani R. All costs, no benefits: how the US-Jordan free trade agreement affects access to medicines. Journal of Generic Medicines. 2009; 6:206-17.
17. Muzaka V. Developing countries and the struggle on the access to medicines front: victories won and lost. Third World Quarterly. 2009; 30:1343-61.
18. Moir HV, Tenni B, Gleeson D, Lopert R. Assessing the impact of alternative patent systems on the cost of health care: the TPPA and HIV treatment in Vietnam. In Asia-Pacific Innovation Conference, University of Technology Sydney 2014 Nov 27 (pp. 27-29).
19. Pascual F. Intellectual property rights, market competition and access to affordable antiretrovirals. Antivir Ther. 2014; 19 Suppl 3 :57-67.
20. Sakthivel S. Access to Essential Drugs and Medicines. In: Lal PE, editor. Background papers on Financing and delivery of Health Care Services in India. New Delhi: Cirrus Graphics Private Limited; 2005. p. 185–212. Copy available from authors.
21. Smith RD, Correa C, Oh C. Trade, TRIPS, and pharmaceuticals. The Lancet. 2009; 373 :684-91.
22. Stavropoulou C, Valletti T. Compulsory licensing and access to drugs. The European Journal of Health Economics. 2015;16:83-94.
23. Supperamaniam M, Alavi R, Gee LH. The Implications of TRIPS to the Pharmaceutical Sector and Access to Medicine: Malaysian Experience. Asian J. WTO & Int'l Health L & Pol'y. 2009; 4:225.
24. Townsend B, Gleeson D, Lopert R. The Regional Comprehensive Economic Partnership, intellectual property protection, and access to medicines. Asia Pacific Journal of Public Health. 2016; 28:682-93.
