# Supplementary material for: Impacts of intellectual property provisions in trade treaties on access to medicine in low and middle income countries: a systematic review
Source: Global Health. 2019 Dec 30;15:88. doi: 10.1186/s12992-019-0528-0 (PMC6937733; doi:10.1186/s12992-019-0528-0)
Supplement: Supplementary file 4 — Additional file 4. Checklist to identify study limitations. [file 12992_2019_528_MOESM4_ESM.docx]

**File name (Additional file 4.doc)**

**Checklist to identify study limitations**

| Objective/research question | Is the research question/aim of the study clearly stated? |
| --- | --- |
|  | Is the importance of the research question stated? |
| Methodology/research design | Is population/comparator   of the study clearly defined? |
|  | Is the type of analysis clearly explained? |
|  | Is the rationale for chosen analysis type provided? |
|  | Is the chosen analysis type adequate/best way to address the research question? |
|  | Is outcome variable clearly identified? |
|  | Are predictor variables selected appropriately? |
| Data | Are the data sources mentioned clearly? |
|  | Is data publicly available? |
|  | Is the selected data appropriate to address the research question? |
|  | Is the chosen time horizon of the data adequate to measure relevant effects of variables? |
|  | Are variables measured in appropriate units? |
|  | Is the sampling strategy relevant to address the research question? |
|  | Is the sampling representative of the target population? |
|  | Is there any sampling bias? |
| Results/findings | Are results/findings discussed adequately? |
|  | Are results/findings appropriate to address the research question? |
|  | Are the results/findings interpreted as correlation or causation or neither? |
|  | Does conclusion follow from the results/finding? |
